# Training in EUS-Guided Fine Needle Aspiration: Safety and Diagnostic Yield of Attending Supervised, Trainee-Directed FNA from the Onset of Training 

**DOI:** 10.1155/2011/378540

**Published:** 2011-11-24

**Authors:** Gregory A. Coté, Christine E. Hovis, Cara Kohlmeier, Tarek Ammar, Abed Al-Lehibi, Riad R. Azar, Steven A. Edmundowicz, Daniel K. Mullady, Hannah Krigman, Lourdes Ylagan, Michael Hull, Dayna S. Early

**Affiliations:** ^1^Division of Gastroenterology, Department of Medicine, Washington University in St. Louis, St. Louis, MO 63110, USA; ^2^Division of Gastroenterology, Department of Medicine, Indiana University School of Medicine, Indianapolis, IN 46202, USA; ^3^Department of Pathology, Washington University in St. Louis, St. Louis, MO 63110, USA

## Abstract

*Background*. The optimal time to initiate hands-on training in endoscopic ultrasound fine needle aspiration (EUS-FNA) is unclear. We studied the feasibility of initiating EUS-FNA training concurrent with EUS training. *Methods*. Three supervised trainees were instructed on EUS-FNA technique and allowed hands-on exposure from the onset of training. The trainee and attending each performed passes in no particular order. During trainee FNA, the attending provided verbal instruction as needed but no hands-on assistance. A blinded cytopathologist assessed the adequacy (cellularity) and diagnostic yield of individual passes. Primary outcomes compared cellularity and diagnostic yield of attending versus fellow FNA passes. *Results*. We analyzed 305 FNA sites, including pancreas (51.2%), mediastinal/upper abdominal lymph node (LN) (28.5%) and others (20.3%). The average proportion of fellow passes with AC was similar to attending FNA—pancreas: 70.3 versus 68.8%; LN: 79.0 versus 81.7%; others 65.5 versus 68.7%; *P* > 0.05); these did not change significantly during the training period. Among cases with confirmed malignancy (*n* = 179), the sensitivity of EUS-FNA was 78.8% (68.4% fellow-only versus 69.6% attending only). There were no EUS-FNA complications. *Conclusions*. When initiated at the onset of EUS training, attending-supervised, trainee-directed FNA is safe and has comparable performance characteristics to attending FNA.

## 1. Introduction

Expertise in endoscopic ultrasound (EUS) requires the development of both cognitive and technical proficiency. The American Society of Gastrointestinal Endoscopy (ASGE) recommends a minimum of 150 total supervised procedures, 75 of which have a pancreatobiliary indication, and 50 cases of fine needle aspiration (FNA) (25 of which are pancreatic FNA) before competency can be determined [1]. These guidelines are based on limited studies of the learning curve in EUS imaging [2–4]. 

FNA represents a common diagnostic maneuver during EUS. Although it is a relatively safe intervention [5], the diagnostic accuracy of FNA in nontrainees improves with greater experience [6, 7]. Mertz and Gautam demonstrated a consistent improvement in the sensitivity of EUS-FNA of pancreatic lesions through 30–40 FNA cases for a single endoscopist who did not undergo dedicated training in EUS [7]. Eloubeidi and Tamhane tracked the diagnostic accuracy and safety of FNA in a single endoscopist who had performed >300 EUS during a dedicated fourth year training program [6]. During the first 300 FNAs performed after training, the diagnostic accuracy did not improve over time, but fewer passes per lesion were required, suggesting continued learning after a high volume training experience. 

A society position statement argues that “successful and safe FNA requires competence in standard EUS imaging” [8] despite no data from advanced endoscopy fellowships in the US evaluating the optimal time to commence hands-on training in EUS-FNA. By incorporating the technical aspects of hands-on training in EUS-FNA early in the cognitive training period, fellows *may* develop competency in this particular skill set earlier in their development. Therefore, we sought to describe the safety and diagnostic yield of attending-supervised EUS-FNA performed by advanced endoscopy fellows at the onset of their training. 

## 2. Methods

### 2.1. Overview

Each year, the Division of Gastroenterology at Washington University in St. Louis trains 1-2 individuals in advanced endoscopic procedures; these advanced fellows had already completed a traditional three-year gastroenterology fellowship but had no prior formal training in EUS. Our practice is to introduce both cognitive and technical aspects of EUS training from the start of the fellowship. Specifically, fellows are instructed on EUS-FNA technique and begin hands-on exposure to this maneuver (i.e., the fellow performs FNA) at the beginning of training, in order to maximize their hands-on training in EUS-FNA during their advanced fellowship. From July, 2008 through June, 2010, we prospectively collected data on the safety and accuracy of attending-supervised EUS-FNA performed by fellows. The study was approved by the Washington University in St. Louis Human Research Protection Office.

### 2.2. FNA Technique

We prospectively enrolled EUS procedures involving FNA that were performed by an attending physician along with a fellow in a combined EUS/cytology database during the study period. We excluded those cases that did not include FNA or those in which the attending performed the entire procedure. In addition, FNA of cystic lesions was excluded, since most of these procedures involve one needle pass. During all cases of EUS-FNA, fellows and their supervising attending were each asked to perform an equal or near-equal number of passes but in no particular order. 

At our institution, cytotechnologists attend EUS-FNA cases and prepare slides, which they then transport to an attending cytopathologist. Therefore, without a preliminary assessment on site, our standard practice is to perform 6-7 passes from solid lesions and 3-4 passes from lymph nodes [9]. However, the total number of passes per lesion is left to the discretion of the attending. All FNA procedures were performed using a curvilinear array echoendoscope (GF-UC140P, Olympus America, Center Valley, Pa, USA). The lesion of interest could have been identified by the fellow with or without attending assistance or by the attending alone. During fellow-directed FNA, the supervising attending may have assisted with positioning of the endoscope, but the FNA was entirely performed by the fellow. The degree of attending assistance in identifying the lesion and positioning the echoendoscope for FNA was not recorded, but in all cases of fellow-directed FNA, the trainee inserted the FNA needle into the lesion and completed the process of tissue acquisition for that pass without any hands-on assistance from the supervising attending. FNA was performed using a 22 or 25 gauge needle (EchoTip Ultra, Cook Medical, Bloomington, Inn, USA). For all passes (fellow or attending directed), the use of suction and a stylet was left to the discretion of the attending endosonographer [10].

### 2.3. Protocol

Fellows recorded which passes they performed (passes were numbered consecutively). Each pass was reviewed by a cytopathologist to assess for (1) adequacy, classified as yes or no and (2) diagnosis (classified as negative, atypical, suspicious, or positive for malignancy). The cytopathologist was blinded to which passes were made by the attending and which by the fellow. Per standard practice in our endoscopy unit, all patients were contacted via telephone 3-4 days after EUS to assess for delayed complications; if the patient could not be reached by telephone, then a postcard was mailed with contact information.

### 2.4. Objectives

Our primary objectives were twofold: (1) to evaluate complication rates of FNA when performed by a supervised fellow and (2) to compare the performance characteristics of EUS-FNA between the attendings and fellows. The first performance characteristic is *specimen adequacy*, determined by a blinded cytopathologist. The second characteristic is the proportion of cases where *at least one* pass would have confirmed a diagnosis of malignancy; this outcome was limited to those cases where malignancy was confirmed either by cytology or a minimum of six months of followup after the EUS. We sought to compare the frequency of achieving at least one diagnostic specimen between groups. A diagnostic specimen was defined as a definitive cytologic diagnosis of malignancy. Using this criterion for diagnosis, we also compare the per-site sensitivity of supervised fellow-FNA compared to attending FNA. Finally, we report the learning curve for each fellow, defined as the average proportion of adequate specimens per FNA site over the course of their training period (one year).

### 2.5. Statistical Analysis

We classified each EUS-FNA site as a separate event for the purpose of this analysis. For each FNA site, passes were grouped by the performing endoscopist: fellow or attending. We report overall complication rates for FNA as simple proportions. For each FNA site, we calculated a percent specimen adequacy for the fellow and attending by dividing the number of adequate FNA passes by the total number of FNA passes performed by that individual. We then compare the average proportion of adequate specimens (a.k.a., specimen adequacy) between groups using one-way analysis of variance (ANOVA). Dichotomous variables (e.g., proportions) were compared using Chi square tests. Statistical analyses were performed using Stata v. 11.0 (StataCorp LP, College Station, Tex, USA).

## 3. Results

During the study period, 2,688 EUS procedures were performed; 38 EUS-FNA sites were excluded due to missing data adjudicating fellow versus attending passes. Of the remaining cases, 305 (11%) EUS-FNA sites met our study criteria, with EUS-FNA being performed by both an attending and a fellow and a cytopathologist study worksheet being completed. There were no complications associated with EUS-FNA. The indications for EUS-FNA and mean number of passes per site performed by the fellow and attending are summarized in Table 1. The majority of sites were pancreatic lesions (51.2%) and mediastinal or upper abdominal lymph nodes (28.5%). The average number of FNA passes per case by both attending and trainee were 6.3 ± 1.5 (pancreas), 5.2 ± 1.9 (lymph node) and 5.9 ± 1.4 (all others) (*P* = 0.007). All three fellows performed a similar number of passes/site.

The proportion of passes that yielded adequate cellularity according to cytopathologist interpretation was similar between fellows and attendings, irrespective of the FNA site (Table 2). Specifically, 66%–79% of fellow FNA passes had adequate cellularity as compared to 69%–82% of attending FNA passes (*P* > 0.05 for each site: pancreas, lymph node, other). A malignant diagnosis was ultimately confirmed in 179 (58.3%) cases. In this subgroup having a confirmation of malignancy, the proportion of cases where the fellow achieved at least one diagnostic FNA pass was similar to the attending, irrespective of location (Table 2). When considering all cases of “suspicious” or “highly suspicious” for malignancy as negative, the overall sensitivity of EUS-FNA was 78.8% in this series; using fellow-FNA passes alone, the sensitivity would have been 68.4% as compared to 69.6% for attending-FNA passes (*P* = 0.82). 

**Figure 1:**
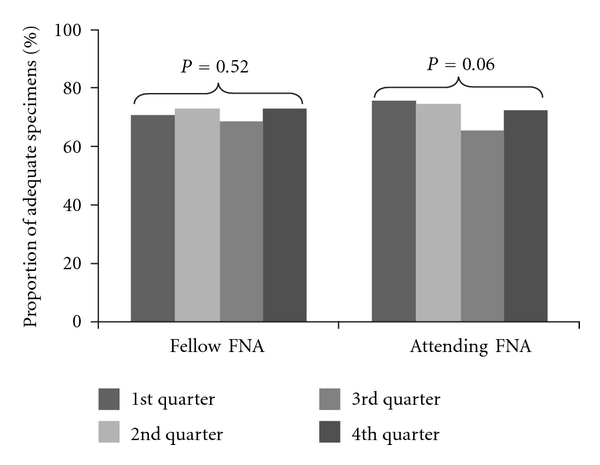
Change in the average proportion of adequate specimens during the training year.

The proportion of fellow EUS-FNA passes with adequate cellularity did not significantly change throughout the training period (Figure 1). During the first quarter of the training period, 70.5% of fellow passes yielded adequate tissue for analysis as compared to 73.2% during the fourth (*P* = 0.52, test of independence).

## 4. Discussion

We present findings from the first prospective study of attending supervised EUS-FNA by advanced endoscopy fellows. Our study demonstrates that fellow-directed EUS-FNA can be performed safely and effectively from the onset of training in diagnostic EUS. 

Based on limited data and expert opinion, the ASGE recommends 150 supervised cases, 75 of which involve pancreatobiliary indications and 50 with EUS-FNA before a formal assessment of competency can be performed [1]. There are no definitive recommendations mandating a fourth year of training in EUS although a survey of GI fellowship directors suggests many 3-year and advanced fellows complete their training with fewer procedures than currently suggested [11, 12]. A survey of 191 endosonographers at a conference on EUS reported a highly variable training experience ranging from self-instruction to observation to hands-on training [13]. Based on limited retrospective data, physicians who have undergone advanced training in pancreatobiliary EUS appear to have greater proficiency with EUS-FNA at the onset of training compared to those who have not [14]. 

Studies investigating the learning curve of EUS-FNA are limited to the experiences of junior faculty who have completed limited [7] or more extensive hands-on training in EUS [6]. Both of these studies demonstrate increased proficiency with greater volume, even in the case of Eloubeidi et al., where the subject/endosonographer had completed 316 supervised EUS procedures, 82 of which involved EUS-FNA during his training period. 

Under attending supervision, the accuracy of fellow EUS-FNA, defined as specimen adequacy, achieving at least one diagnostic sample, and sensitivity, is comparable to attending FNA and does not change during the training period. We assume that the amount of attending guidance during fellow FNA diminishes as the competency of the fellow steadily improves; our study was not designed to quantify this. Our results describe the technical component of EUS-FNA; that is, the fellow can follow instruction from their supervisor and complete the hands-on aspects of FNA at the onset of training. Our study was not designed to comment on the progression of the cognitive aspects of EUS and EUS-FNA during the training period. Since the degree of attending intervention during each FNA was not recorded, we cannot determine if or when the trainee was able to independently perform EUS-FNA. 

Fellows are not capable of independently performing EUS-FNA from the start; rather, our results show that the presence of attending supervision assures comparable adequacy when the fellow performs FNA versus attending-directed FNA. Therefore, allowing fellow-directed FNA earlier in the training period maximizes the number of supervised cases without compromising patient safety and the performance characteristics (sensitivity/specificity) of EUS-FNA. By considering all “suspicious” samples as negative, our reported sensitivity of EUS-FNA (78.8%) is consistent with previous studies [15–18]. The optimal approach to training in EUS remains unclear, but our findings suggest early hands-on exposure to EUS-FNA is safe and does not reduce the sensitivity of FNA for confirming a diagnosis of malignancy. Animal models and endoscopic simulators may further shorten the learning curve for trainees by providing early hands-on experience, combining advances in the technical and cognitive aspects of EUS [19–22]. 

## 5. Conclusions

Attending-supervised, fellow-directed EUS-FNA from the onset of training in EUS is safe and feasible. By allowing early hands-on exposure to EUS-FNA, the fellow maximizes their experience with this technique before embarking on an independent career as an endosonographer. In addition to studying other technical and cognitive benchmarks in EUS, future studies should focus on the learning curve of achieving independent EUS-FNA.

## Figures and Tables

**Table 1:** Procedure characteristics.

Variable	Fellow A	Fellow B	Fellow C	Total number of cases	*P* value
	(*n* = 112)	(*n* = 99)	(*n* = 94)		
Location (%)					
(i) Pancreas	59 (52.7)	54 (54.6)	43 (45.7)	156 (51.2)	0.55^†^
(ii) Mediastinal/upper abdominal lymph node	29 (25.9)	25 (25.2)	33 (35.1)	87 (28.5)	
(iii) All others	24 (21.4)	20 (20.2)	18 (19.2)	62 (20.3)	

Fellow FNA passes/site (mean ± SD)	3.2 ± 1.1	2.8 ± 1.2	3.0 ± 1.1	N/A	0.873^‡^
Attending FNA passes/site (mean ± SD)	2.7 ± 1.2	3.3 ± 1.4	2.8 ± 1.5	N/A	0.02^‡^

^†^Pearson's test of independence.

^‡^One-way analysis of variance.

**Table 2:** Performance characteristics of supervised fellow-FNA and attending-FNA.

Variable	Fellow*	Attending**	*P* value
Average proportion of FNA passes with adequate cellularity (±SD)			
(i) Pancreas	70.3 ± 32.4	68.8 ± 35.6	0.71
(ii) Mediastinal/upper abdominal lymph node	79.0 ± 34.1	81.7 ± 33.7	0.61
(iii) All others	65.5 ± 37.5	68.7 ± 38.8	0.65

Proportion of cases with at least one diagnostic specimen (95% confidence interval) (*n* = 179 cases)			
(i) Pancreas	70.2 (61.0, 79.4)	71.2 (62.1, 80.4)	0.87
(ii) Mediastinal/upper abdominal lymph node	57.8 (43.4, 72.2)	62.2 (48.1, 76.4)	0.67
(iii) All others	78.1 (63.8, 92.4)	75.0 (60.0, 90.0)	0.77

Variables are expressed as the average proportion ± SD, per site.

*Fellow denotes the cumulative data from all three participating EUS fellows.

**Attending FNA v. all fellow FNA.
